# Clinical Impact of Small Bowel Capsule Endoscopy in Obscure Gastrointestinal Bleeding

**DOI:** 10.3390/medicina56100548

**Published:** 2020-10-19

**Authors:** Ana-Maria Singeap, Camelia Cojocariu, Irina Girleanu, Laura Huiban, Catalin Sfarti, Tudor Cuciureanu, Stefan Chiriac, Carol Stanciu, Anca Trifan

**Affiliations:** 1 Department of Gastroenterology, Faculty of Medicine, “Grigore T. Popa” University of Medicine and Pharmacy, 700115 Iasi, Romania; anamaria.singeap@yahoo.com (A.-M.S.); gilda_iri25@yahoo.com (I.G.); huiban.laura@yahoo.com (L.H.); cvsfarti@gmail.com (C.S.); drcuciureanutudor@gmail.com (T.C.); stefannchiriac@yahoo.com (S.C.); ancatrifan@yahoo.com (A.T.); 2 Institute of Gastroenterology and Hepatology, “St. Spiridon” Emergency Hospital, 700111 Iasi, Romania; stanciucarol@yahoo.com

**Keywords:** small bowel capsule endoscopy, obscure gastrointestinal bleeding, overt obscure gastrointestinal bleeding, iron deficiency anemia, angioectasia, diagnostic yield

## Abstract

*Background and objectives:* The most frequent indications for small bowel capsule endoscopy (SBCE) are obscure gastrointestinal bleeding (OGIB) and iron deficiency anemia (IDA). The aim of this study was to evaluate the diagnostic yield (DY) of SBCE in overt and occult OGIB, as well as its impact on the clinical outcome. *Materials and Methods:* This study retrospectively included all cases of OGIB investigated by SBCE in a tertiary care referral center, between 1st January 2016 and 31st December 2018. OGIB was defined by overt or occult gastrointestinal bleeding, with negative upper and lower endoscopy. Occult gastrointestinal bleeding was either proved by a fecal test or presumptively incriminated as a cause for IDA. DY was defined as the detection rate for what were thought to be clinically significant findings. DYs for overt and occult bleeding were assessed and compared. Gender, age, hemoglobin levels, NSAID consumption and the use of anticoagulants were recorded. Following SBCE results, individual therapeutic decisions were made, and follow-up data were recorded. *Results:* 224 SBCE examinations were performed for OGIB, of which 148 were for overt OGIB, and 76 for unexplained IDA. Positive findings were found in 139 patients, resulting in an overall DY for OGIB of 62%, higher in overt OGIB (75%) compared to IDA (37%). The most frequent findings were small bowel angioectasias (62.2% in overt OGIB and 78.5% in IDA). On multivariate logistic regression analysis, only hemoglobin level <10 g/dL and anticoagulants were the variables independently associated with positive findings. All patients received medical, endoscopic or surgical treatment and had good clinical outcome during follow-up. *Conclusion:* SBCE has a high diagnostic yield and a positive impact on management of patients with OGIB.

## 1. Introduction

The advent of small bowel capsule endoscopy (SBCE) has revolutionized non-invasive direct visualization of the small bowel, considered until then the “black box” of the gastrointestinal (GI) tract. SBCE brought progress in the management of small bowel diseases, especially obscure gastrointestinal bleeding (OGIB), Crohn’s disease and small bowel tumors, as well as NSAID-induced enteritis, hereditary polyposis syndromes and complicated celiac disease. The most frequent indication for SBCE remains OGIB, either overt or occult [1]. Overt OGIB is represented by clinically manifest hematochezia or melena with no cause identified after upper and lower endoscopy, whereas the current understanding of occult bleeding consists in either a positive fecal occult blood test or chronic iron deficiency anemia (IDA). SBCE has become the first-line investigation in the diagnosis of both OGIB and IDA [2]. The diagnostic performance of SBCE in OGIB/IDA is superior to other diagnostic modalities (push enteroscopy, small bowel barium radiography, CT angiography, CT enterography, MR enterography) [3,4]. When SBCE was compared to double balloon enteroscopy, a similar diagnostic accuracy for OGIB was reported [5]. In clinical practice, the performance of SBCE is formulated in terms of diagnostic yield (DY), understood as the detection rate for what are thought to be clinically significant findings [2]. Studies showed an overall DY of SBCE for OGIB of around 60%, with higher DYs in patients with active bleeding compared to those with occult bleeding, and in cases explored sooner (until 14 days) after the bleeding episode [6,7]. Occult bleeding is either defined by a positive fecal test or implied by IDA. Current guidelines recommend complete assessment of IDA before SBCE, in order to eliminate non-hemorrhagic causes [8]. In our study, we aimed to evaluate the DY of SBCE in overt and occult OGIB, as well as the etiological spectrum of OGIB and the impact of SBCE results on clinical management and outcome.

## 2. Materials and Methods

### 2.1. Patients

Our study retrospectively included all cases of OGIB investigated by SBCE in a tertiary referral center between 1st January 2016 and 31st December 2018.

The local Ethical Committee approved this study (approval ID 15/03.06.2019). Written informed consent was obtained from each patient and the study was conducted according to the Declaration of Helsinki.

### 2.2. Methods

The entire diagnostic work-up was conducted following the current guidelines for the use of SBCE.

OGIB was defined by overt or occult gastrointestinal bleeding, with negative upper endoscopy and colonoscopy. Occult gastrointestinal bleeding was either proved by a fecal test or presumptively incriminated as a cause for IDA (defined as hemoglobin <13 g/dL in men and <12 g/dL in women). Anamnesis was rigorously conducted in order to identify other potential explanations for IDA (gynecological causes for women) or risk factors for bleeding (associate pathology, risk from ongoing medications such as NSAIDs, anticoagulants and antiplatelet agents). Before SBCE, celiac disease was excluded for all patients with unexplained IDA. In some cases of overt bleeding, a second-look endoscopy was performed in order to reassure the true negative result of the first exam. A second-look colonoscopy was also performed in selected cases, the reasons for repeating the investigations being the incomplete preparation and/or the lack of evidence that the prior exam was complete. This analysis included only patients with isolated IDA, with no other symptoms or signs that could have suggested inflammatory bowel disease in the first place.

SBCE exams were performed after contraindications (mainly suspicion of intestinal obstruction) were excluded and after the patient signed the informed consent, which contained all information needed for fully understanding the procedure and the potential complications, retention risk included. Second- and third-generation endoscopic capsules for small bowel examination (PillCam SB2 and PillCam SB3) manufactured by Given Imaging, Yoqneam, Israel were used. Examinations were performed after overnight fasting for 12 h. For overt OGIB, all SBCE were performed within 14 days of a bleeding episode. They were all performed in an inpatient setting, and the results were interpreted by a single trained specialist. Patients were allowed to drink clear fluid 2 h after the capsule was swallowed and a light lunch 4 h later. Evaluation of the relevance of the lesions was made according to Saurin classification [9]. Bleeding lesions and potentially bleeding lesions, as well as fresh blood in the lumen, were considered diagnostic findings. Diagnostic yields for overt and occult bleeding were assessed and compared. Following SBCE results, individual therapeutic decisions were made, and follow-up data were recorded. The observational interval ranged between 18 months for the procedures performed by the end of the study period and 54 months for the procedures performed at the beginning of the study period.

### 2.3. Statistical Analysis

Statistical analysis was carried out using the SPSS 17.0 software (SPSS Inc., Chicago, IL, USA). Continuous variables with normal distribution are expressed as mean ± SD, non-normal variables are expressed as median and interquartile range. Categorical variables are expressed as absolute values and percentages. Univariate analysis was performed for each recorded variable, and variables with a *p*-value < 0.1 were included in multivariate analysis (logistic regression) in all patients to identify the predictive factors of positive diagnosis by SBCE [10]. Odds ratio (OR) with 95% confidence interval (CI) was calculated (for qualitative variables included in the logistic regression). A *p*-value of less than 0.05 was considered statistically significant.

## 3. Results

### 3.1. Patients, Indications and Prior Work-Up

There were 224 SBCE examinations for OGIB, representing almost two thirds (65.6%) of the total number of SBCEs performed in the specified period (341 for all indications). The median patient age was 53 years old, and there were more females (64.2%). One hundred and fifty patients (67%) were older than 50 years. One hundred and four patients (46.4%) were on oral anticoagulant treatment (vitamin K antagonists, with international normalized ratio (INR) within therapeutic range, or non-vitamin K oral anticoagulants), or antiplatelet treatment (like aspirin, clopidogrel, ticagrelor). The most frequent associated conditions requiring risk medication were cardiovascular and/or neurological pathologies; other comorbidities were respiratory, renal, liver or rheumatological disease. The mean hemoglobin level was 9.8 ± 1.8 g/dL (range 4.8–11.7) and 60 patients (26.7%) had received blood transfusion (Table 1). One hundred and forty-eight patients were investigated for overt OGIB, and 76 for unexplained IDA, either with proved occult bleeding by fecal test (22 patients), or with suspected occult gastrointestinal bleeding with negative or not available fecal test (54 patients). The 148 patients with overt OGIB initially presented with either melena or hematochezia. They were either admitted in emergency settings or referred after primary management by other departments or centers, mainly of secondary care. They were all investigated after hemodynamic stabilization by upper gastrointestinal endoscopy and colonoscopy, with no evidence of potentially bleeding lesions. Thirty-two of them had also a second-look upper endoscopy, justified by the suboptimal examination in emergency conditions; none of the second examinations came with a positive diagnosis. Ten patients had also a second-look colonoscopy, mainly justified by incomplete preparation during the first examination, and no lesions were found. The SBCE completion rate, defined as visualization of the cecum, was 95%.

### 3.2. Findings and Diagnostic Yield

Significant findings, summarized in Table 2, were found in 139 cases out of all 224 examinations for OGIB, resulting in an overall DY of 62%, higher in overt OGIB (75%) than in occult OGIB (37%). By far, the most frequent findings were small bowel angioectasias (62.2% of patients with overt OGIB, and 78.5% of patients with IDA). Other causes for overt OGIB and unexplained IDA were small bowel tumors, Crohn’s disease and NSAID-induced enteritis. One of the cases with unexplained IDA, with no findings at SBCE, was later diagnosed with atransferinemia and referred to a hematological unit. One case of retention was registered in a patient with radiation enteritis, solved surgically. The retention rate was 0.44%.

On univariate analysis, age >50 years, NSAID use, anticoagulants/antiplatelet drugs and hemoglobin level <10 g/dL were associated with positive findings in patients with OGIB and IDA, whereas on multivariate logistic regression analysis, only hemoglobin level <10 g/dL and anticoagulants/antiplatelet therapy were the variables independently associated with positive findings (Table 3).

### 3.3. Impact on Patient Management and Outcome

Following SBCE, individual decisions were made (Table 4). Most patients with angioectasias had argon plasma coagulation by enteroscopy or were surgically treated in a few cases due to multiple small bowel angioectasias; interventional radiology with selective embolization during arteriography was performed in one case. The sole patient with cecum angioectasia was treated by argon plasma coagulation during the subsequent colonoscopy. An interclinic approach was followed for all patients with concomitant hemorrhagic risk medication, for treatment revision. Knowing that there is no unique standard diagnostic test for Crohn’s disease, all patients with findings consistent with small bowel Crohn’s disease were complementarily explored in order to add other arguments favoring the diagnosis. Medical treatment was initiated with good clinical evolution. All cases with small bowel tumors were fully investigated afterwards. Enteroscopy was performed to fully characterize tumors and CT enterography or MR enterography were used for stadialization. The patients were referred for surgical treatment, the majority of tumors being either stromal gastro-intestinal tumors or adenocarcinomas; the sole exception was one case of intestinal lymphoma, which was referred to the hematological unit. The patient with parasitosis received specific antiparasitary treatment, with resolution of anemia. In the case of suspected NSAID-induced enteritis, revision of treatment was made, with no further intervention needed. The cases with fresh blood seen at SBCE were referred to further work-up, and they were afterwards diagnosed with angioectasias (two cases) and small bowel tumors (two cases), with proper subsequent interventional or surgical management. Apart from the case of retention, SBCE had less impact on clinical management for patients of radiation enteritis; they were further conservatively managed.

## 4. Discussion

SBCE has become the first line investigation for suspected small bowel pathologies, and OGIB is its most frequent indication. In terms of SBCE indication, the two notions—occult OGIB and IDA—are overlapping. In turn, reciprocally, IDA could be classified as IDA with proved occult OGIB, and IDA with suspected occult OGIB, knowing that a negative fecal test does not exclude occult bleeding. In this study, we did not include the cases with suspected “non-bleeding” anemia, such as suspected celiac disease or other malabsorption syndromes. Before considering SBCE, all alternative possible causes for IDA were looked for. Even so, a diagnosis of atransferinemia was made after SBCE work-up was completed. The rarity of these cases exculpates this, but it is undoubtedly of utmost importance to perform an exhaustive diagnostic work-up before SBCE.

Defining SBCE performance parameters in terms of sensitivity and specificity is difficult due to the fact that there is no gold standard applicable. The appearance itself of SBCE is the result of the need to better explore the small bowel. Even if until the advent of SBCE, the small bowel had its own methods of being investigated, they were imperfect, and efforts were made to overcome their shortcomings. SBCE proved itself to be more useful as a diagnostic tool than almost all the other methods [8,11]. SBCE was compared to intraoperative enteroscopy as gold standard, but this is not a method currently applicable [12]. Two meta-analyses have shown that SBCE is superior to radiological imaging and push enteroscopy for diagnosing OGIB, while it has a comparable DY to double balloon enteroscopy [13,14]. Therefore, because sensitivity and specificity are unsuitable performance parameters for SBCE, a surrogate parameter is used, namely the DY. It is defined as the detection rate for what are thought to be clinically significant findings, reported for all investigations performed. The DY takes into account all the investigations with clinically significant findings, even if they are not necessarily directly related to the indication of SBCE. In our study, all SBCE findings were related to the indication of administration (OGIB or IDA). Therefore, we can affirm that, in this study, the calculated DY truly reflected the SBCE diagnostic performance. The global DY of 62% is similar to other studies in the literature [13,14] and even higher for the cases of overt OGIB (DY = 75%). The DY in IDA cases was 36%, and could be considered modest; as discussed above, an influence factor for the performance of SBCE could be the selection of cases. Some studies reported that increased age may predict a higher diagnostic yield with SBCE [15], a characteristic not confirmed by our study. Another reported factor which may predict the ability of SBCE to detect small bowel pathology in OGIB (including IDA) was the use of anticoagulants [4,16], which was confirmed by our study.

The most frequent findings were angioectasias, confirming the results of other studies [17,18,19]. They are recognized as potentially bleeding lesions, especially if concomitant anticoagulant or antiplatelet medication is taken. Their identification by SCBE is diagnostic, but in almost all cases there is no active bleeding seen, so their responsibility is circumstantial. Angioectasia could be in fact innocent bystander, the more so for the cases of IDA without evidence of occult bleeding. Questions and doubts regarding the real cause of IDA could be raised in these cases. On the other hand, if we do consider angioectasia as a true cause, another scenario could be valid: the false negative results in anemia, by the paleness of angioectasia corresponding to the low level of hemoglobin. We found that hemoglobin level was an independent predictive factor of positive diagnosis by SBCE.

Our study showed a good diagnostic performance of SBCE for diagnosing small bowel tumors, confirming previous reports [20,21,22,23], but it must be emphasized that SBCE cannot offer the final diagnosis. Additional investigations are required in order to have a histological diagnosis, a precise localization and a complete stadialization. Enteroscopy could be considered a complementary exam, while other type of investigations (CT enterography, MR enterography) will provide additional information needed before therapeutic decision. The small bowel tumors are probably the most illustrative for the dual portrait of SBCE nowadays: the great advantage of non-invasiveness is counterbalanced by the disadvantage of the incapability of taking biopsies. However, this important limit of SBCE is not far from being overcome by the next generation of SBCE with diagnostic and therapeutic capabilities [24].

Even if the lesion itself was not found in the four cases of active bleeding, the presence of the fresh blood in the bowel was considered diagnostic. This is a well-accepted principle, given that a positive diagnostic of small bowel bleeding is made; SBCE is able to guide further investigations.

The retention rate in our study was 0.44%, remarkably low compared to other studies performed for other indications, such as known or suspected Crohn’s disease (2.6%) or small bowel tumors (2.1%) [25,26]. However, we confirmed that patients with OGIB are at the lowest risk for capsule retention [27].

SBCE had a favorable impact on the evolution of the majority of patients, showing that, even if it is not a therapeutic modality, it has its own value as a safe, non-invasive diagnostic method. The place of SBCE in current guidelines is already justly stated, as a first choice in suspected small bowel pathologies. However, SBCE must be wisely chosen during the patient work-up, with utmost attention paid to patient selection and appropriate referral to further investigation or therapeutic techniques.

Our study has some strengths, such as a representative number of patients both with OGIB and IDA, but also several limitations, such as being a single-center study and the lack of long-term follow up for all patients.

## 5. Conclusions

SBCE has good performance parameters for OGIB and proved itself as a safe technique. SBCE has a high diagnostic yield and a positive impact on the management of patients with OGIB. The overall DY was 62%, higher in cases of overt OGIB (75%) and lower for unexplained IDA (37%). Subsequent individualized management assured a good outcome for all the diagnosed cases. SBCE remains a visual technique, and small bowel tumor discovery had to be followed by supplementary work-up. Small bowel angioectasias were the dominant findings. Additional tests should follow negative SBCE exams. Regardless of diagnosis, the SBCE orientated the subsequent management and had a favorable clinical impact in terms of immediate and long-term outcome. Despite its actual limits, such as the lack of taking biopsies and of therapeutic valences—which will most likely be overcome in the near future—SBCE has an indisputable diagnostic role in small bowel pathologies, having on its side the advantage of being a non-invasive and safe procedure.

## Figures and Tables

**Table 1:** Patient demographics, clinical and laboratory parameters.

All Patients, *n*	224
Gender, male/female, *n*	80/144
Age (years), median (IQR)	53 (16)
NSAID consumption, *n* (%)	129 (58.6)
Oral anticoagulants (NOAC, VKA)/antiplatelet drugs, *n* (%)	104 (NOAC, *n* = 11, VKA, *n* = 36; antiplatelet drugs, *n* = 57) (46.4)
Hemoglobin, g/dL (mean ± SD)	9.8 ± 1.8
Blood transfusion, *n* (%)	60 (26.7)

NOAC: non-vitamin K oral anticoagulants; VKA: vitamin K antagonists.

**Table 2:** Significant findings at small bowel capsule endoscopy (SBCE) in overt obscure gastrointestinal bleeding (OGIB) and unexplained iron deficiency anemia (IDA).

Patients with OGIB (*n* = 224)	Findings	Cases, *n* (%)
Overt OGIB (*n* = 148) Positive SBCE, *n* = 111 Negative SBCE, *n* = 37	Small bowel angioectasias	69 (62.2%)
	Small bowel tumors	18 (16.2%)
	Crohn’s disease	10 (9%)
	NSAID-induced enteritis	5 (4.5%)
	Radiation enteritis	4 (3.6%)
	Fresh blood	4 (3.6%)
	Cecum angioectasia	1 (0.9%)
Unexplained IDA (*n* = 76) Positive SBCE, *n* = 28 Negative SBCE, *n* = 48	Angioectasias	22 (78.5%)
	Crohn’s disease	3 (10.7%)
	Small bowel tumors	1 (3.6%)
	Parasitosis	1 (3.6%)
	Lymphoma	1 (3.6%)

**Table 3:** Univariate and multivariate regression analysis of risk factors associated with positive findings in patients with OGIB and IDA.

Parameter	Univariate Analysis			Multivariate Analysis				
	OR	95%CI	*p*-Value	OR	95%CI	*p*-Value	β cof	Wald
Anticoagulant/antiplatelet drug use	6.73	3.78–11.27	**0.002**	2.27	1.63–5.19	**0.018**	1.328	7.998
Hemoglobin < 10 g/dL	7.28	3.11–14.27	**0.010**	1.94	1.06–4.79	**0.037**	2.551	9.12
Age > 50 years	2.26	1.15–5.27	**0.028**	1.43	0.32–5.37	**0.499**	0.413	0.456
Male gender	2.97	1.47–6.88	**0.047**	1.76	0.29–10.44	0.534	0.565	0.387
NSAID use	3.55	2.01–7.38	**0.035**	1.61	0.98–23.11	**0.067**	1.564	3.769

Bold font indicates statistical significance.

**Table 4:** Interventions and outcome after SBCE.

Diagnosis at SBCE	No of Cases	Intervention Type (*n*)	Outcome (*n*)
Angioectasias	92	Endoscopic therapy (52) Surgery (2) Radiological therapy (1) Medical therapy (37)	No further bleeding (70), correction of anemia (16)
Crohn’s disease	13	Specific medical treatment	No further bleeding (10), correction of anemia (2)
Small bowel tumors	19	Surgery	No further bleeding, correction of anemia
Small bowel lymphoma	1	Hematological referral	Persistence of anemia
Parasitosis	1	Specific medical treatment	Correction of anemia
NSAID enteritis	5	NSAID withdrawal, iron supplementation treatment	No further bleeding
Radiation enteritis	4	Medical treatment (3) Surgery for retention (1)	No further bleeding (2)
Fresh blood	4	Further work-up Surgery (2), endoscopic treatment (2) after final diagnosis	No further bleeding (4)

## References

[1] Liu K., Kaffes A.J. (2011). Review article: The diagnosis and investigation of obscure gastrointestinal bleeding. Aliment. Pharmacol. Ther..

[2] Pennazio M., Spada C., Eliakim R., Keuchel M., May A., Mulder C.J., Rondonotti E., Adler S.N., Albert J., Baltes P. (2015). Small-bowel capsule endoscopy and device-assisted enteroscopy for diagnosis and treatment of small-bowel disorders: European Society of Gastrointestinal Endoscopy (ESGE) Clinical Guideline. Endoscopy.

[3] Triester S.L., Leighton J.A., Leontiadis G.I., Fleischer D.E., Hara A.K., Heigh R.I., Shiff A.D., Sharma V.K. (2005). A meta-analysis of the yield of capsule endoscopy compared to other diagnostic modalities in patients with obscure gastrointestinal bleeding. Am. J. Gastroenterol..

[4] Koulaouzidis A., Rondonotti E., Giannakou A., Plevris J.N. (2012). Diagnostic yield of small-bowel capsule endoscopy in patients with iron-deficiency anemia: A systematic review. Gastrointest. Endosc..

[5] Pasha S.F., Leighton J.A., Das A., Harrison M.E., Decker G.A., Fleischer D.E., Sharma V.K. (2008). Double-balloon enteroscopy and capsule endoscopy have comparable diagnostic yield in small-bowel disease: A meta-analysis. Clin. Gastroenterol. Hepatol..

[6] Singh A., Marshall C., Chaudhuri B., Okoli C., Foley A., Person S.D., Bhattacharya K., Cave D.R. (2013). Timing of video capsule endoscopy relative to overt obscure GI bleeding: Implications from a retrospective study. Gastrointest. Endosc..

[7] Yung D.E., Rondonotti E., Giannakou A., Avni T., Rosa B., Toth E., Lucendo A.J., Sidhu R., Beaumont H., Ellul P. (2017). Capsule endoscopy in young patients with iron deficiency anaemia and negative bidirectional gastrointestinal endoscopy. United Eur. Gastroenterol. J..

[8] Enns R.A., Hookey L., Armstrong D., Bernstein C.N., Heitman S.J., Teshima C., Leontiadis G.I., Tse F., Sadowski D. (2017). Clinical Practice Guidelines for the Use of Video Capsule Endoscopy. Gastroenterology.

[9] Saurin J.C., Delvaux M., Gaudin J.L., Fassler I., Villarejo J., Vahedi K., Bitoun A., Canard J.M., Souquet J.C., Ponchon T. (2003). Diagnostic value of endoscopic capsule in patients with obscure digestive bleeding: Blinded comparison with video push-enteroscopy. Endoscopy.

[10] Sambataro G., Giuffrè M., Sambataro D., Palermo A., Vignigni G., Cesareo R., Crimi N., Torrisi S.E., Vancheri C., Malatino L. (2020). The Model for Early COvid-19 Recognition (MECOR) Score: A Proof-of-Concept for a Simple and Low-Cost Tool to Recognize a Possible Viral Etiology in Community-Acquired Pneumonia Patients during COVID-19 Outbreak. Diagnostics.

[11] Segarajasingam D.S., Hanley S.C., Barkun A.N., Waschke K.A., Burtin P., Parent J., Mayrand S., Fallone C.A., Jobin G., Seidman E.G. (2015). Randomized controlled trial comparing outcomes of video capsule endoscopy with push enteroscopy in obscure gastrointestinal bleeding. Can. J. Gastroenterol. Hepatol..

[12] Hartmann D., Schmidt H., Bolz G., Schilling D., Kinzel F., Eickhoff A., Huschner W., Moller K., Jakobs R., Reitzig P. (2005). A prospective two-centre study comparing wireless capsule endoscopy with intraoperative enteroscopy in patients with obscure GI bleeding. Gastrointest. Endosc..

[13] Chen X., Ran Z.H., Tong J.L. (2007). A meta-analysis of the yield of capsule endoscopy compared to double-balloon enteroscopy in patients with small-bowel disease. World J. Gastroenterol..

[14] Brito H.P., Ribeiro I.B., de Moura D.T.H., Bernardo W.M., Chaves D.M., Kuga R., Maahs E.D., Ishida R.K., de Moura E.T.H., de Moura E.G.H. (2018). Video capsule endoscopy vs double-balloon enteroscopy in the diagnosis of small-bowel bleeding: A systematic review and meta-analysis. World J. Gastrointest. Endosc..

[15] Sidhu R., Sanders D.S., Kapur K., Leeds J.S., McAlindon M.E. (2009). Factors predicting the diagnostic yield and intervention in obscure gastrointestinal bleeding investigated using capsule endoscopy. J. Gastrointest. Liver Dis..

[16] Olano C., Pazos X., Avendano K., Calleri A., Ketzoian C. (2018). Diagnostic yield and predictive factors of findings in small-bowel capsule endoscopy in the setting of iron-deficiency anemia. Endosc. Int. Open.

[17] Penazzio M., Santucci R., Rondonotti E., Abbiati C., Beccari G., Rossini F. (2004). Outcome of patients with obscure gastrointestinal bleeding after capsule endoscopy. Report of 100 consecutive cases. Gastroenterology.

[18] Ell C., Remke S., May A., Helou L., Henrich R., Mayer G. (2002). The first prospective controlled trial comparing wireless capsule endoscopy with push enteroscopy in chronic gastrointestinal bleeding. Endoscopy.

[19] Redondo-Cerezo E., Perez-Vigara G., Perez-Sola A., Gómez-Ruiz C.J., Chicano M.V., Sánchez-Manjavacas N., Morillas J., Perez-Garcia J., Garcia-Cano J. (2007). Diagnostic yield and impact of capsule endoscopy on management of patients with gastrointestinal bleeding of obscure origin. Dig. Dis. Sci..

[20] van Turenhout S.T., Jacobs M.A., van Weyenberg S.J., Herdes E., Stam F., Mulder C.J., Bouma G. (2010). Diagnostic yield of capsule endoscopy in a tertiary hospital in patients with obscure gastrointestinal bleeding. J. Gastrointest. Liver Dis..

[21] Cobrin G.M., Pittman R.H., Lewis B.S. (2006). Increased diagnostic yield of small-bowel tumors with capsule endoscopy. Cancer.

[22] Trifan A., Singeap A.M., Cojocariu C., Sfarti C., Stanciu C. (2010). Small-bowel tumors in patients undergoing capsule endoscopy: A single center experience. J. Gastrointest. Liver Dis..

[23] Rondonotti E., Pennazio M., Toth E., Menchen P., Riccioni M.E., De Palma G.D., Scotto F., De Looze D., Pachofsky T., Tacheci I. (2008). European Capsule Endoscopy Group; Italian Club for Capsule Endoscopy (CICE); Iberian Group for Capsule Endoscopy. Small-bowel neoplasms in patients undergoing video capsule endoscopy: A multicenter European study. Endoscopy.

[24] Singeap A.M., Stanciu C., Trifan A. (2016). Capsule endoscopy: The road ahead. World J. Gastroenterol..

[25] Singeap A.M., Trifan A., Cojocariu C., Sfarti C., Stanciu C. (2011). Outcomes after symptomatic capsule retention in suspected small-bowel obstruction. Eur. J. Gastroenterol. Hepatol..

[26] Liao Z., Gao R., Xu C., Li Z.S. (2010). Indications and detection, completion, and retention rates of small-bowel capsule endoscopy: A systematic review. Gastrointest. Endosc..

[27] Hoog C.M., Bark L.A., Arkani J., Gorsetman J., Broström O., Sjöqvist U. (2012). Capsule retentions and incomplete capsule endoscopy examinations: An analysis of 2300 examinations. Gastroenterol. Res. Pract..

